# Wideband LTE Power Amplifier with Integrated Novel Analog Pre-Distorter Linearizer for Mobile Wireless Communications

**DOI:** 10.1371/journal.pone.0101862

**Published:** 2014-07-17

**Authors:** Eswaran Uthirajoo, Harikrishnan Ramiah, Jeevan Kanesan, Ahmed Wasif Reza

**Affiliations:** Department of Electrical Engineering, Faculty of Engineering, University of Malaya, Kuala Lumpur, Malaysia; Gazi University, Turkey

## Abstract

For the first time, a new circuit to extend the linear operation bandwidth of a LTE (Long Term Evolution) power amplifier, while delivering a high efficiency is implemented in less than 1 mm^2^ chip area. The 950 µm × 900 µm monolithic microwave integrated circuit (MMIC) power amplifier (PA) is fabricated in a 2 µm InGaP/GaAs process. An on-chip analog pre-distorter (APD) is designed to improve the linearity of the PA, up to 20 MHz channel bandwidth. Intended for 1.95 GHz Band 1 LTE application, the PA satisfies adjacent channel leakage ratio (ACLR) and error vector magnitude (EVM) specifications for a wide LTE channel bandwidth of 20 MHz at a linear output power of 28 dBm with corresponding power added efficiency (PAE) of 52.3%. With a respective input and output return loss of 30 dB and 14 dB, the PA’s power gain is measured to be 32.5 dB while exhibiting an unconditional stability characteristic from DC up to 5 GHz. The proposed APD technique serves to be a good solution to improve linearity of a PA without sacrificing other critical performance metrics.

## Introduction

Information and communication technology (ICT) plays a significant role in globalization process which has reorganized the world economy [1]. Therefore, communication standards need to be continuously upgraded to the next level. This has led into the birth of Long Term Evolution (LTE) protocol. LTE is a prominent solution to fulfill the continuous demand for high data rate transmission. LTE is capable in establishing downlink peak data rates up to 326.4 Mbps and 86.4 Mbps for the uplink [2]. It also has an advantage of variable channel bandwidth, which ranges from 1.4 MHz to 20 MHz. Service providers are expected to benefit from this luxury, in the context of cost and channel allocations. LTE utilizes single carrier frequency division multiple access (SC-FDMA) for uplink and orthogonal frequency division multiple access (OFDMA) for downlink as its multicarrier modulation standard catering towards high data rates transmission [3]. SC-FDMA has a comparable performance and complexity to OFDMA, with an advantage of lower peak-to- average-power-ratio (PAPR) [4]. Typically, the PAPR of OFDMA signal is 10 dB whereas for SC-FDMA is 7 dB [2].

In order to transmit signals with high PAPR linearly (without any clipping), power amplifiers are usually operated at backed off output power. For example, if the signal’s PAPR is 7 dB, the PA’s maximum output power has to be 35 dBm in order to meet LTE linearity specifications (ACLR and EVM) at an output power of 28 dBm. This degrades its efficiency, η. η is defined as:
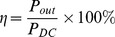
(1)where P_out_ represents the output power delivered by the PA whereas P_DC_ denotes the DC power supplied to the PA. The relationship between backed off output power and efficiency is expressed as [5]:



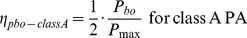
(2)

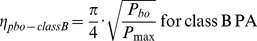
(3)where P_bo_ and P_max_ represent backed off output power and maximum output power respectively. With reference to (2) and (3), PA which transmits LTE signal with PAPR of 7 dB, delivers an efficiency of 9.9% and 35% at backed off output power, P_bo_ of 28 dBm (P_max_ is 35 dBm) if it’s operating in class-A or class-B mode. As a consequence, the battery energy is drained out rapidly.

The solution to improve η at backed off output power is to either reduce the back-off level by reducing the P_max_ (smaller device size) or through the introduction of efficiency enhancement techniques. However, reduction of backed off output power level drives the PA to fail linearity specifications, thus mandating linearization techniques to curb this issue [5]. In a nutshell, efficiency enhancement techniques focus in improving the efficiency of a linear PA, whereas linearization techniques improve the linearity of a highly efficient but non-linear PA.

In recent published works, envelope tracking power amplifier (ETPA) gained a popular platform in efficiency enhancement methodology. In this technique, the supply voltage of the PA is modulated respective to the RF input drive voltage while sustaining a constant load resistance, thus improving the efficiency at low output power. An envelope detector is responsible to track the input drive voltage. Due to the stringent requirement in the linear output power which is specified by the adjacent channel leakage ratio (ACLR) and error vector magnitude (EVM), GaAs HBT is favored in realizing the amplifying core of LTE PA [6]–[7]. Therefore, dual chip solution is often required, which serves to be a burden in context of cost, complexity and size. In order to curb the aforementioned disadvantages, single chip implementation using SiGe BiCMOS process [8] and fully CMOS process [9]–[10] are explored. However, the resulting linear output power is low.

On the other hand the linearization techniques offer analog pre-distortion (APD) and digital pre-distortion (DPD) method which are gaining intense attention in recent reported works. In these methods, the input RF signal to the PA is distorted prior to amplification by changing its non-linear transfer function’s magnitude and phase to inherit an opposite response to the phase and magnitude of the non-linear PA output. The outmost priority is regularly given in cancelling out the third order intermodulation product (IMD3) components which is generated due to amplification of more than single tone input signal. IMD3 tend to generate spectrum re-growth at the adjacent channel located closer to the PA’s operational channel. In the DPD method, these non-linear responses are generated with the aid of a DSP processor [11]–[13]. The complexity in integration, resulting in larger chip area and dual fabrication process are among the visible disadvantages of DPD. On the other hand, APD offers a simple solution of integrating additional active devices, usually within the same process at the input of the power amplifier [14]–[19]. Passive elements are alternately utilized as a pre-distorter, to linearize a non-linear class-E PA [20]. In addition, recent reported works also prioritize in improving the efficiency of the PA by introducing mode locking method with cascode CMOS [21], multi-stage Doherty PA [22] and inductive degeneration technique [23]. Additionally, methods to improve the modeling technique for larger device size to precisely predict thermal performance which affects the efficiency of a PA is also proposed [24].

In this paper, a new technique is introduced in improving the linearity of a LTE PA at reduced back off output power. Mathematical analysis proves that by selecting an optimum conduction angle, the third order components effect which has primary influence on linear transmission can be reduced without degrading the efficiency severely. In order to meet the strict ACLR and EVM specifications for LTE, an analog pre-distorter block is integrated at the input of the main amplifier thus enabling single chip solution. The total size of the chip is 950 µm × 900 µm. This reduces the cost and space required on the phone board. The outline of this work is organized as follows. In Section 2, the principle of operation is presented. The design methodology based on the proposed operation is given in Section 3. In Section 4, with the aid of 16QAM LTE signal of 20 MHz channel bandwidth, the characterized linearity and efficiency performance of the designed PA is provided. Finally, a conclusion is drawn in Section 5.

Mathematically, the illustrated waveform can be represented as:

## Principle of Operation

### Optimum Conduction Angle and Efficiency Analysis

**Figure 1:**
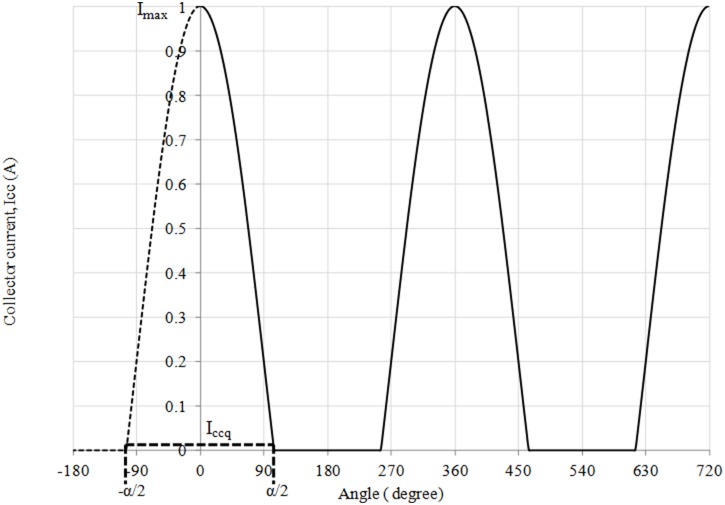
The collector current waveform plot. I_ccq_ is the quiescent biasing point whereas α/2 is the corresponding conduction angle.

The trade-off between linear operation and efficiency is fundamentally determined by the PA’s biasing point, alternately known as the conduction angle of the PA. Selecting an optimum conduction angle is essential in designing a linear and efficient PA. As the conduction angle reduces, the rise of odd and even orders components are more significant. Third order component adversely affects the linearity performance of the PA. In this work, a mathematical analysis is observed to determine the first and third order responses across the conduction angle for a HBT amplifier, in reference to the RF collector current waveform plot in **Figure 1**.

Mathematically, the illustrated waveform can be represented as:



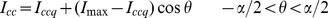
(4)where



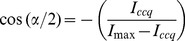
(5)Hence resulting,



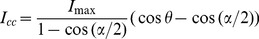
(6)The Fourier analysis is adapted to Equation (6), computing the DC component and magnitude of the n^th^ order components, which are given by Equations (7) and (8), respectively:



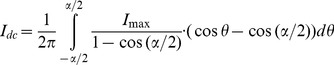
(7)

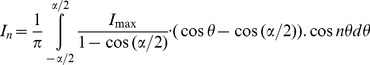
(8)


**Figure 2:**
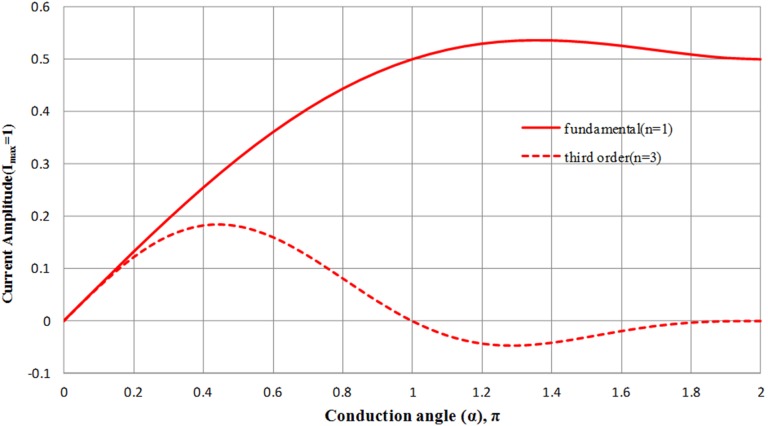
Fundamental and third order current components across conduction angle.

Solving Equation (7) for DC term and Equation (8) for the fundamental component, I_1_ (n = 1) and third order component I_3_ (n = 3) results in:
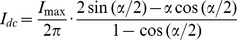
(9)

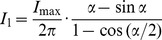
(10)

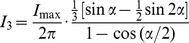
(11)I_1_ and I_3_ are plotted across the conduction angle, as depicted in **Figure 2**.

It can be observed from **Figure 2** that as the conduction angle increases, the fundamental current component increases too whereas the third order current component reduces. An interesting observation is at the conduction angle within the range of π<α<1.8π. In this region, the fundamental component is the highest and the third order component is at the lowest. This shows that it is theoretically possible to obtain higher fundamental output power, although the PA is not biased at class-A (conduction angle 2 π) mode. The lowest third order component on the other hand promises a linear operation to a certain extent without sacrificing the efficiency at much.

In order to determine the efficiency corresponding to α = 2 π which represents the conduction angle of a class-A amplifier, equation (9) and (10) is appreciated, resulting in I_dc_ = I_max_/2 π and I_1_ = I_max_/2 π hence reflecting the efficiency of class A, which is I_1_/I_dc_ to be 1. Using this result, the relationship between the conduction angle and efficiency in reference to class-A mode is given by:
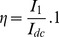
(12)

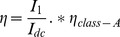
(13)

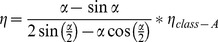
(14)


### Analog pre-distorter (APD) Design

The goal of the linearization technique in this work is to reduce the typical back-off output power level needed to meet the LTE linearity specifications. This is accomplished by integrating an APD block at the input of the low voltage main amplifier.

**Table:** **Table 1.** Performance summary of the PA.

	Results
Technology	2 um InGaP/GaAs HBT
Supply voltage	3.3 V
Frequency	1.95 GHz
Mode	LTE
Channel BW	20 MHz
Max Linear output Power	28 dBm
EVM (16-QAM)	2.36% at 28 dBm
PAE	52.3%
Gain	32.5 dB
S11	–30 dB
S22	–14 dB
Stability	Unconditionally Stable

**Table:** **Table 2.** Performance comparison of published LTE power aplifiers.

Work	OperatingFrequency[GHz]	LTE ChannelBandwidth[MHz]	SupplyVoltage [V]	Gain [d]	Maximum LinearOutput Power[dBm]	PAE [%]	ChipSize[mm^2^]
[6]	2.5	10	3.3	24.8	25.8	31.6	2.6×1.7
[7]	2.5	20	6.0	29.0	30.0	45.0	1×1.6
[8]	2.4	5	4.2	16.0	24.3	42.0	1.1×1.5
[29]	0.84	10	3.4	27.2	27.0	34.5	1.7×1.7
[30]	0.93	10	2.0	28	25.1	15.0	1.8×1.85
*This work*	1.95	20	3.3	32.5	28.0	52.3	0.95×0.9

**Figure 3:**
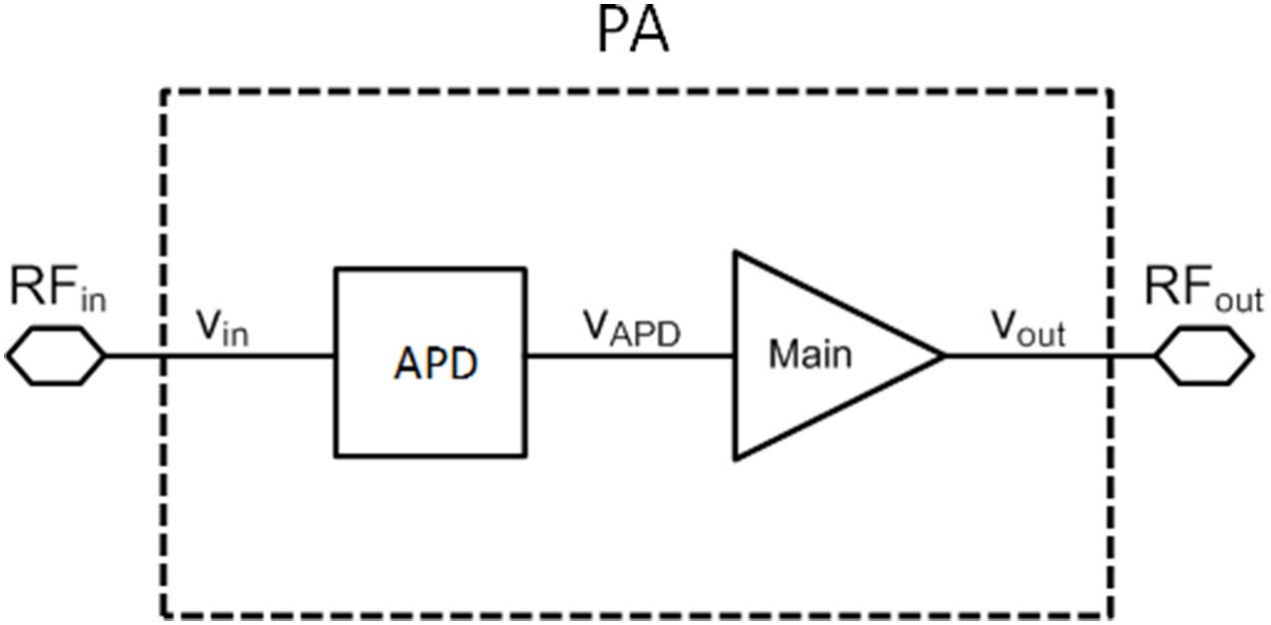
IMD3 cancellation analysis.

Operating the main amplifier at low supply voltage headroom results an early gain compression at low output power. This is due to the rise of the third order intermodulation distortion (IMD3) components as the output power increases. The rise of the third order nonlinear components significantly degrades the ACLR [25]. In order to mitigate this adverse effect, the APD architecture is integrated to produce IMD3 components, which are equal in amplitude but 180° out-of-phase respective to the IMD3 spurs generated by the main amplifier. Thus, the IMD3 cancellation attained extends the overall linear output power span of the PA. The operation in **Figure 3** can be explained with the aid of the following analysis. From the expression of the power series, given by:
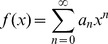
(15)

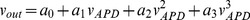
(16)

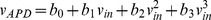
(17)


Taking into consideration of the fundamental and third order components only and incorporating (17) into (16):
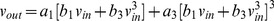
(18)

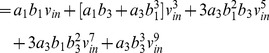
(19)to nullify the third order interaction components,



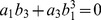
(20)

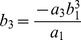
(21)


normalizing (21) in the context of the linear fundamental gain, 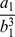, establishes



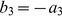
(22)It can be concluded from (22) that in order to achieve IMD3 cancellation, the third order non-linear components generated at the output of the APD need to have an opposite response respective to the third order non-linear components generated by the main amplifier. In practice, this can be achieved if an opposite AM-AM and AM-PM responses are generated between the APD and main amplifier [26].

## Design Methodology

**Figure 4:**
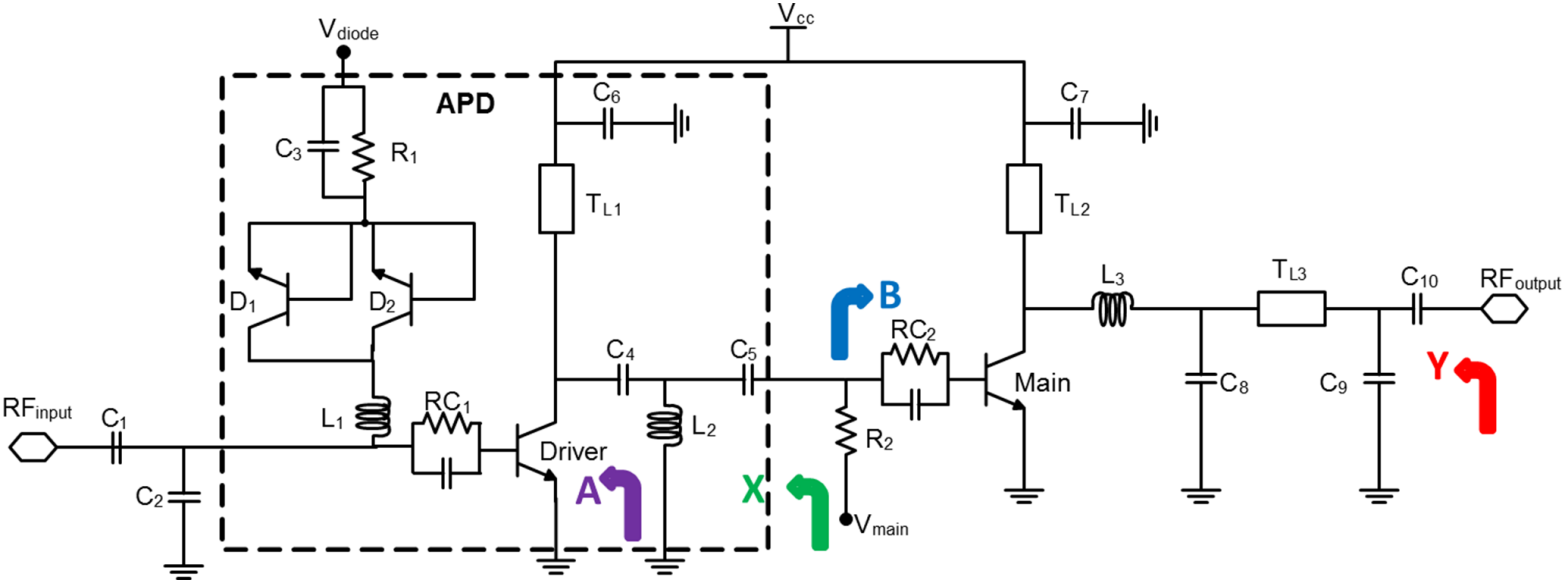
Schematic diagram of the LTE PA with built in APD. X denotes the output impedance of the APD, Y is the output impedance of the main amplifier.

**Figure 4** illustrates the schematic of the proposed LTE PA. Parallel base ballast resistor and capacitor, RC is used to mitigate thermal runaway phenomenon. It also helps to stabilize both amplifying stages, ruling out the need for a feedback circuitry. A dual stage output matching network consisting of L_3_, C_8_, TL_3_ and C_9_ is proposed to transform the 50 ohm load to the desired output impedance of the main amplifier. L_3_ is implemented as bondwires, whereas TL_3_ denotes a transmission line.

The main amplifier is biased at a conduction angle of 1.1π. The selected conduction angle has low IMD3 component as described in **Figure 2**. The optimal trade-off between IMD3 and efficiency results in a PAE of 74.8% based on the theoretical equation governed in (14).

**Figure 5:**
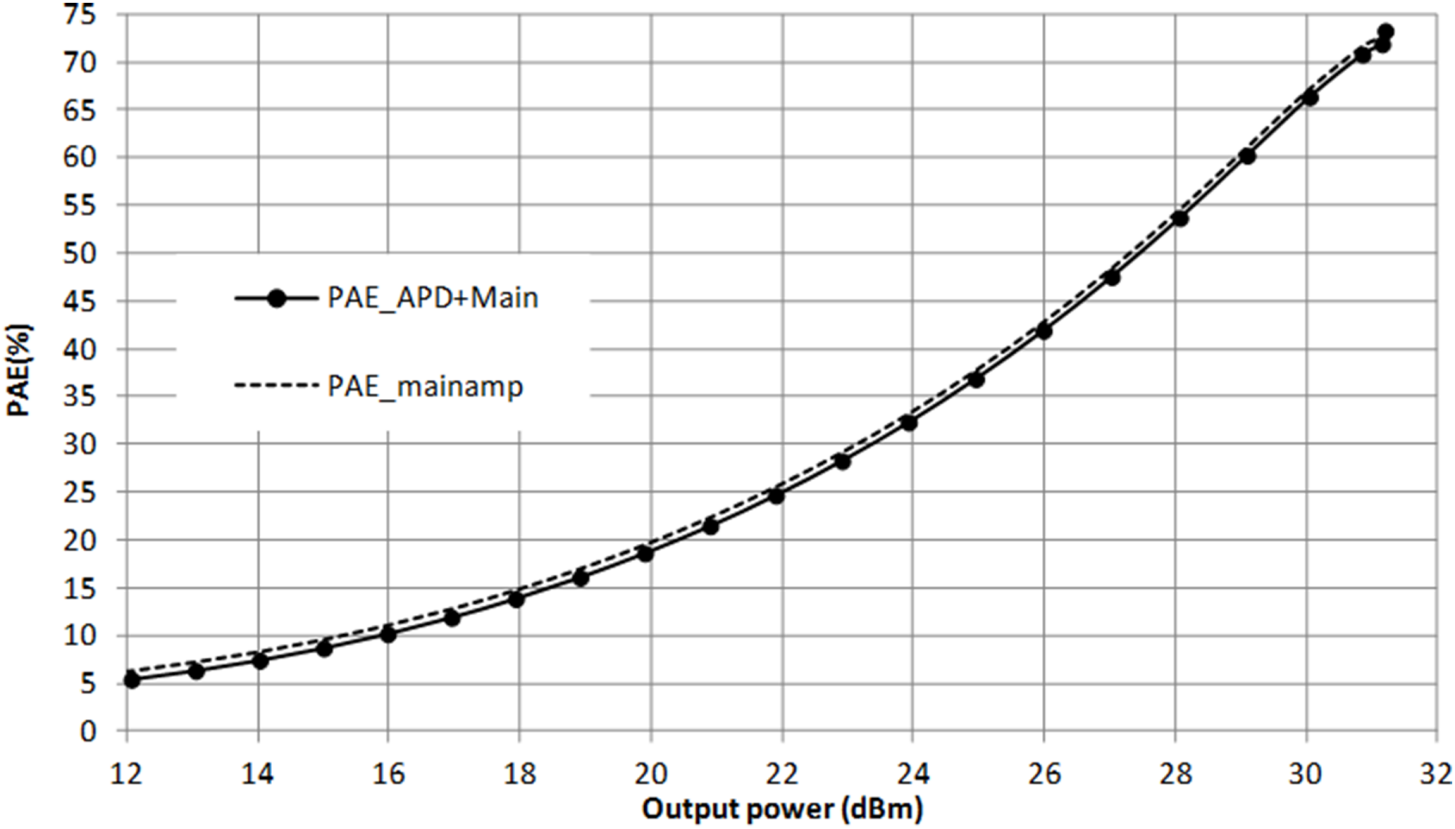
PAE simulation result for the LTE PA with and without the integration of Analog Pre-distorter (APD).

Utilizing the Agilent Advanced Design System (ADS) verification tool, a harmonic balance simulation has been performed to determine the PAE of the proposed circuit in **Figure 4**, in which the PAE is defined as:
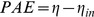
(23)where 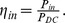 **Figure 5** describes the resulting plot obtained, the PAE of the main amplifier degrades 0.8% as compared to the theoretical value, an acceptable penalty paid by integrating the ballast resistors on each unit cell of the main amplifier to mitigate thermal runaway phenomenon. The PAE drops reasonably further to 73.2% at maximum output power with the integration of the APD.

**Figure 6:**
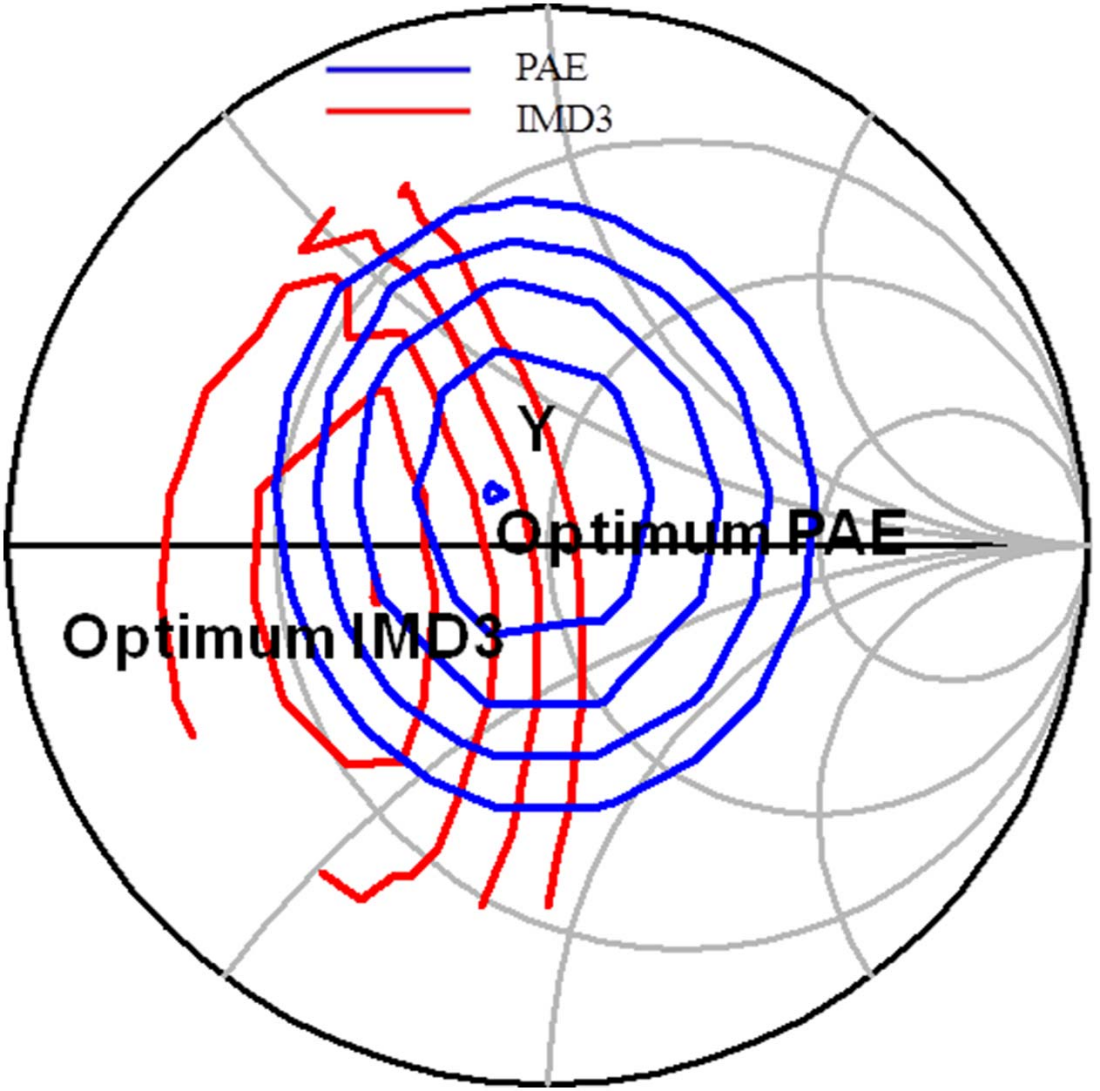
Load pull simulation result illustrating the IMD3 and PAE contours of the main amplifier. The PAE contour is plotted in 1% step whereas the IMD3 contour is plotted in 2 dB step at output power of 28 dBm.

**Figure 6** illustrates the IMD3 and PAE load pull contours prior linearization. Y is the output impedance of the main amplifier. These contours are plotted at an output power of 28 dBm. Despite having an optimum conduction angle of 1.1π, the optimum IMD3 point is still located almost 8 dB away from Y, as described in **Figure 6**.

**Figure 7:**
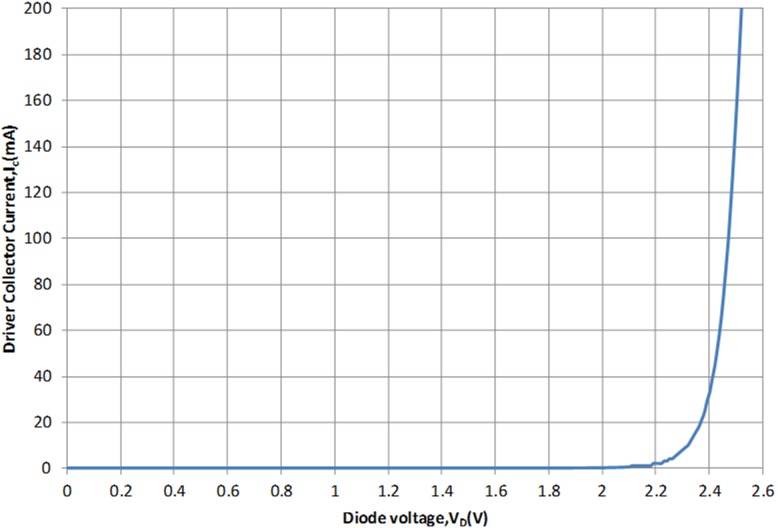
Biasing profile of the integrated parallel base collector diodes. The supply voltage to the diodes has to be at least 2.2

In HBT, spectral re-growth is mainly contributed by its base-collector parasitic capacitance C_bc_ [27]. To mitigate this effect, we propose a novel phase cancellation method by integrating a base collector diode at the input of the driver amplifier. The reverse bias capacitance C_bc-reversebias_ and forward bias capacitance C_bc-forwardbias_ are expressed as follows [28]:
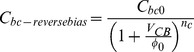
(24)where C_bc0_ is the collector-base capacitance when V_CB_ = 0, φ_0_ is the collector base junction built in voltage and n_c_ is the grading coefficient of the collector base junction. In order to generate an opposite output phase response, the collector-base junction is forward biased. The aforementioned collector base capacitance is expressed by:



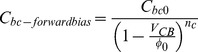
(25)Based on (24) and (25) the positive and negative phase insight in effect to V_CB_ cancels off the C_bc-reversebias_ with single C_bc-forwardbias_. However, with the aid of two base collector diodes (C_bc-forwardbias_ > C_bc-reversebias_) an opposite phase response (AM-PM) is observed at the output of the APD. The biasing profile of the diode to turn ON the driver amplifier in the APD is shown in **Figure 7**.

**Figure 8:**
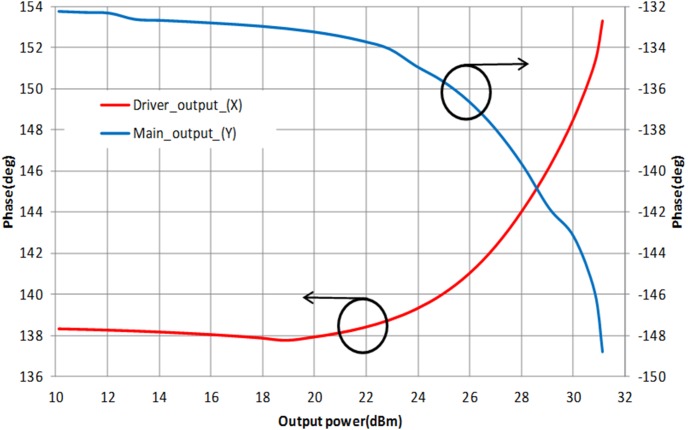
Simulated AM-PM responses of the APD and main amplifier.

The simulated AM-PM responses at the output of the APD and main amplifier are illustrated in **Figure 8**. The driver’s phase expansion and main amplifier’s phase compression cancels out each other contributing to the improvement of the IMD3 performance. R_1_ and C_3_ in **Figure 4** is a bias isolation circuit in which it ensures the bias input does not interfere with the effort on the AM-PM cancellation.

Meanwhile generation of an opposite AM-AM response is accomplished through the T section intermediate matching network consisting of C_4_, L_2_ and C_5_. The Smith plot in.

**Figure 9:**
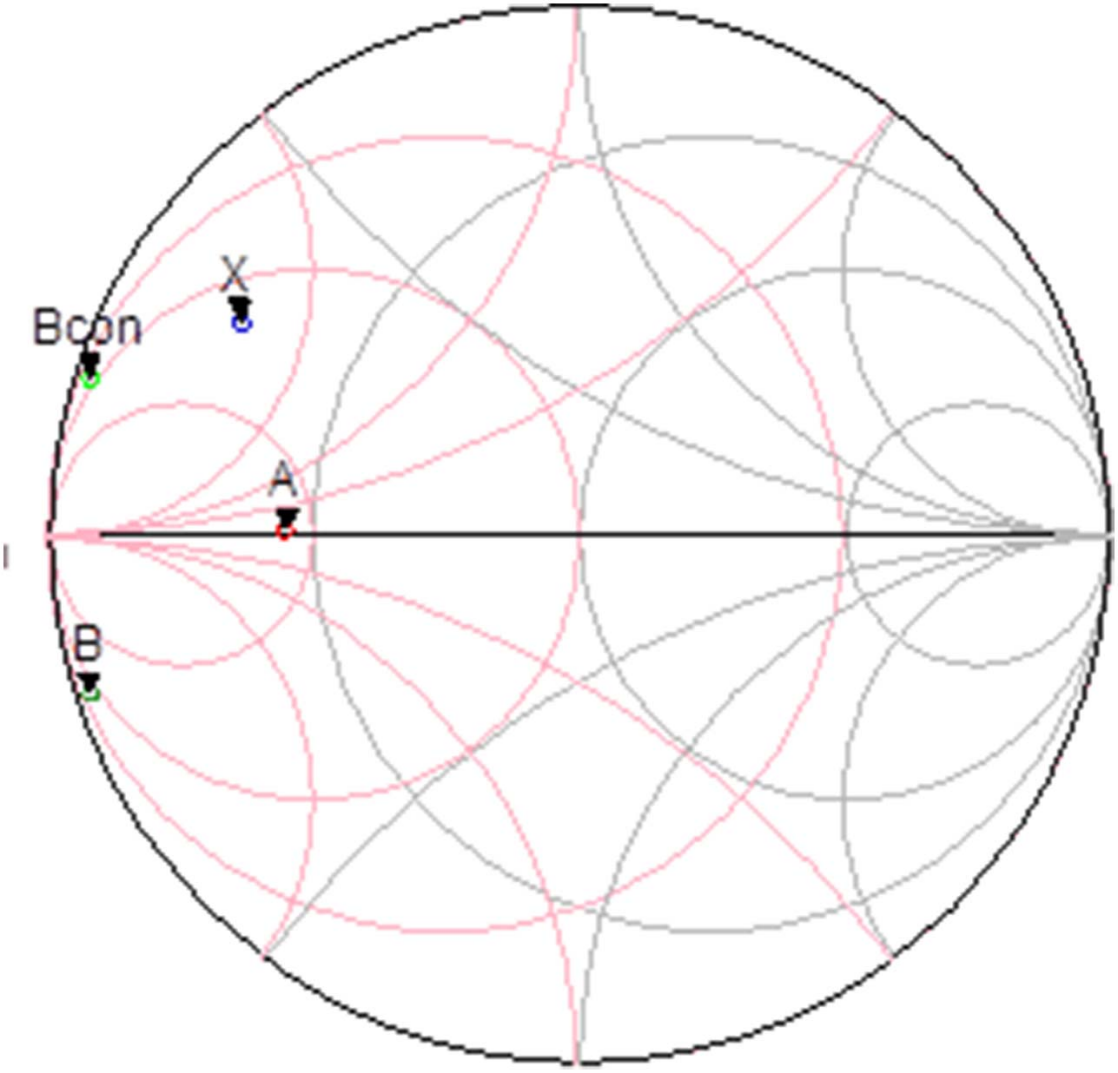
Location of the impedance point of the driver (A), main amplifier (B) and APD(X).

**Figure 10:**
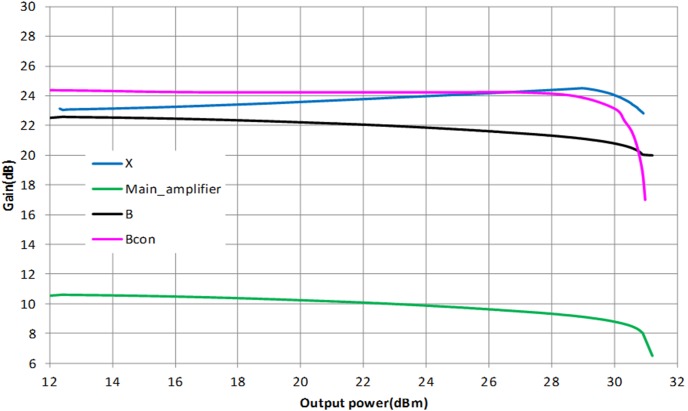
AM-AM profile for various intermediate matching network impedances mentioned in Figure S9.

**Figure 11:**
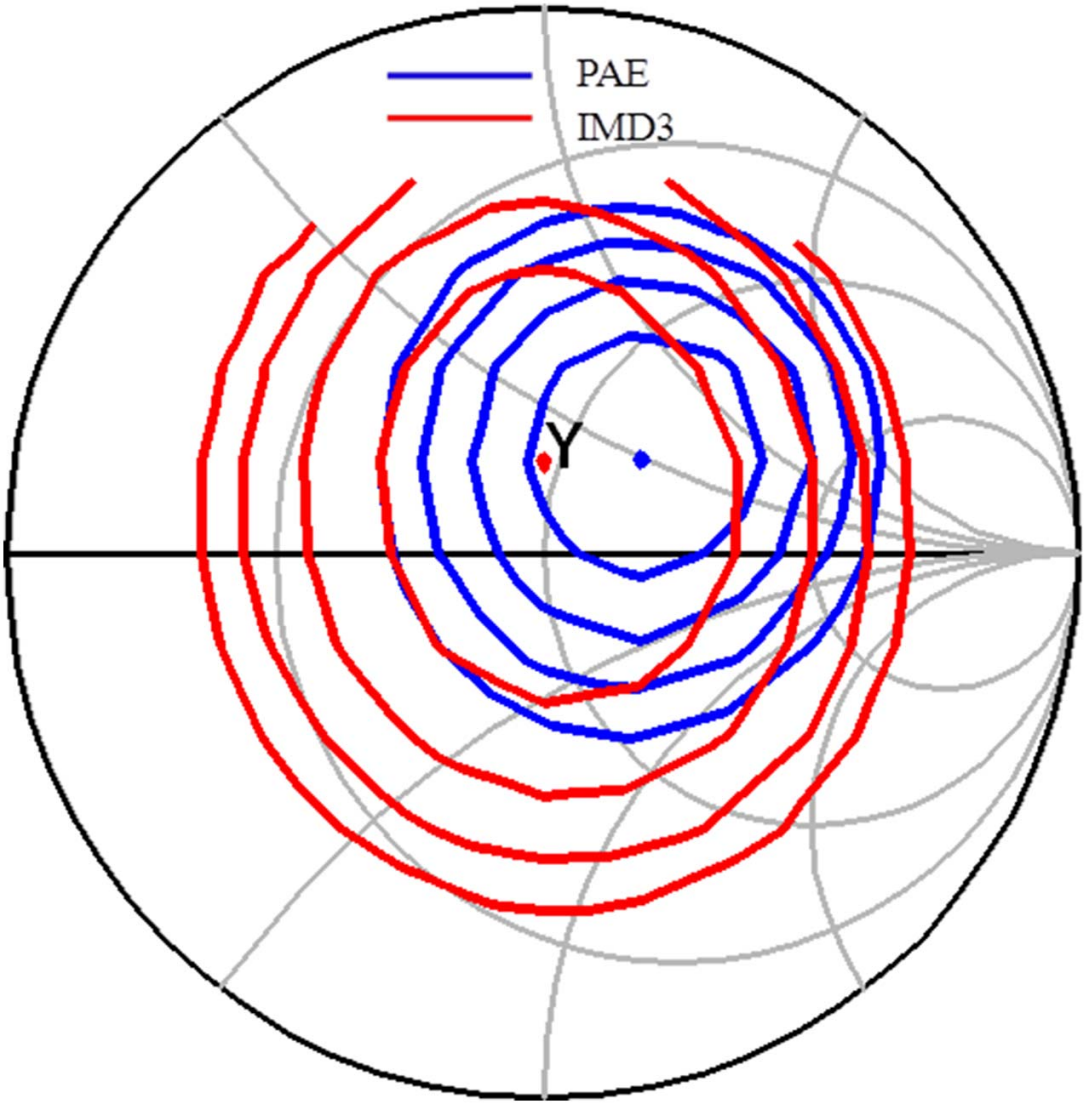
IMD3 and PAE contours of the PA after linearization.

**Figure 9** illustrates the location of the output impedance of the driver denoted at point A. This location is also shown in the schematic in **Figure 4**. Impedance A is potentially matched to X, B or Bcon. Point B describes the input impedance of the main amplifier integrated with an input ballasting parallel RC network. Point X is the output impedance of the APD where else Bcon is the conjugate of B. Based on the profile in **Figure 10**, matching towards point X observes a favorable gain expansion which compensates the gain compression of the main amplifier. Matching towards point B observes a gain compression at a lower output power which would not result into a desirable IMD3 cancellation. Finally, point Bcon observes a flat profile till the 1 dB compression point in which results into a similar effect as with point B matching. The effect of AM-AM and AM-PM cancellation between the APD and main amplifier in the PA is illustrated in **Figure 11**. The IMD3 optimum impedance moves to location Y while the PAE degrades slightly due to the current consumption of the APD.

## Measurement Results

**Figure 12:**
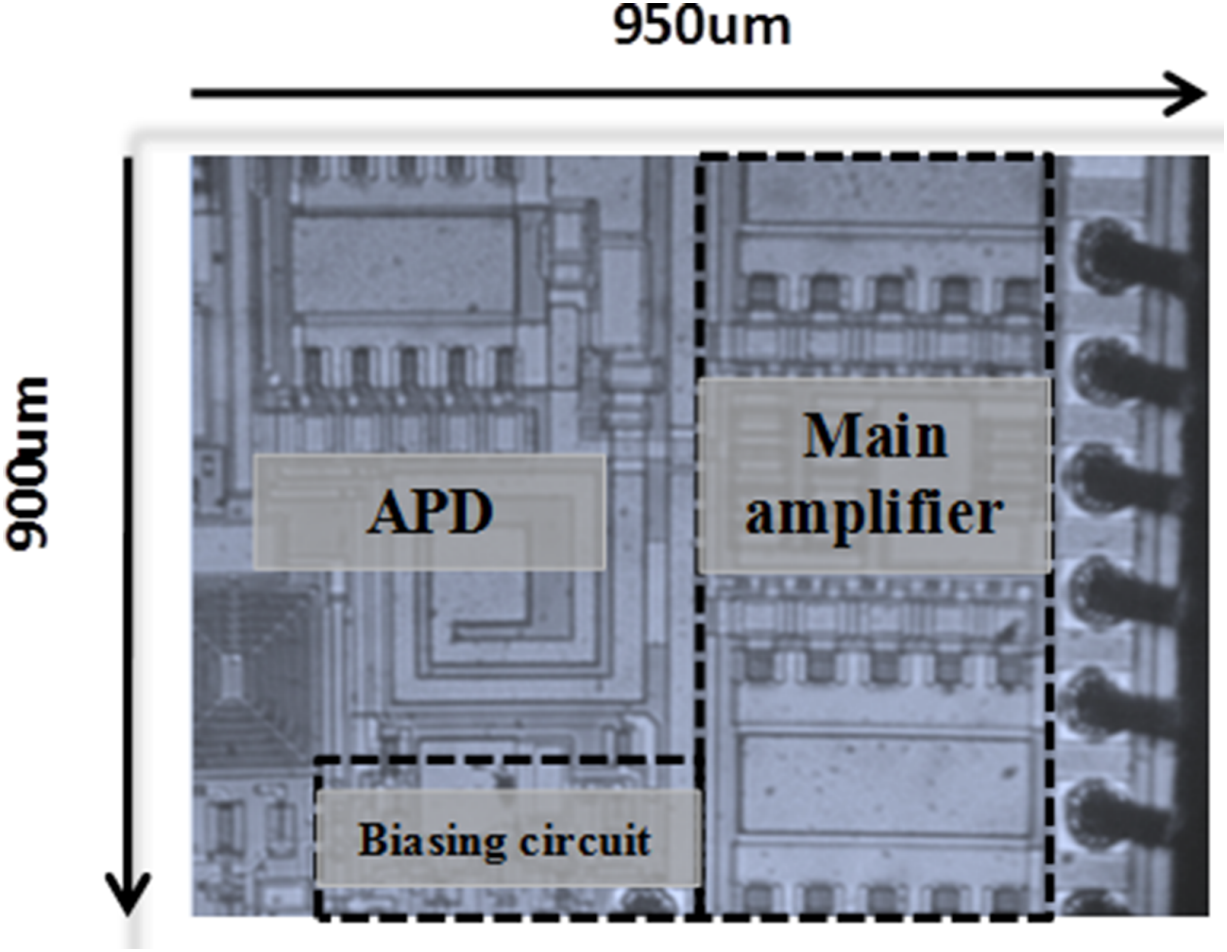
Die microphotograph of the fabricated PA with integrated APD. The size of the die is less than 1× 1 mm.

**Figure 13:**
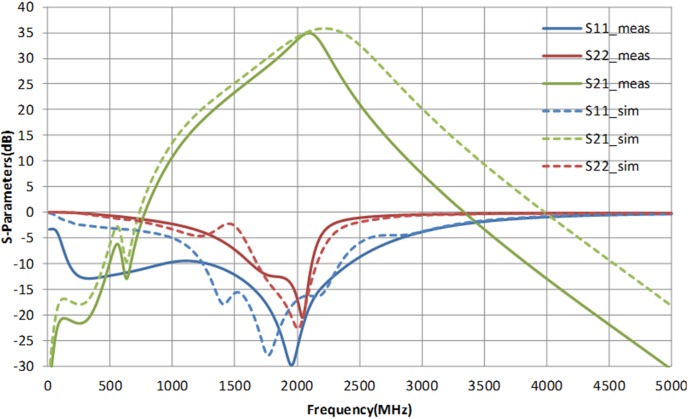
Measured and simulated S-parameters of the PA with supply headroom of 3.3 V.

**Figure 14:**
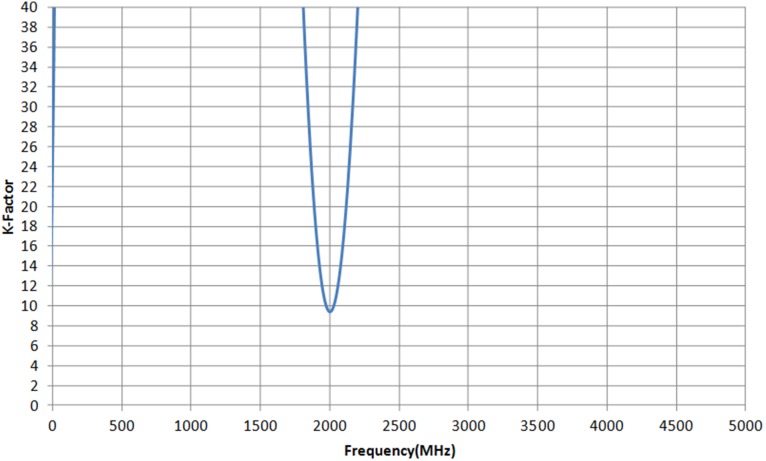
PA has K-Factor >1 from DC up to 5 GHz.

**Figure 15:**
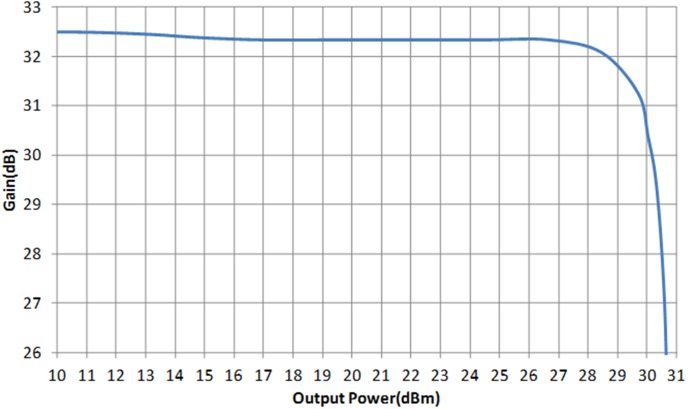
Gain vs output power plot of the PA.

**Figure 12** illustrates the microphotograph of the die, fabricated in 2 µm InGaP/GaAs HBT process. The size of the die is 950 µm × 900 µm. The main amplifier and APD are integrated in a single chip, along with the input matching network. The output matching network is placed externally, on a printed circuit board. The supply voltage headroom of the LTE PA is 3.3 V. The simulated and measured S-parameter of the proposed PA is shown in **Figure 13**. The S_11_ and S_22_ are well matched at 1.95 GHz, with a corresponding gain S_21_ of 32.5 dB. A low S_11_ indicates that the APD does not generate a severe input mismatch loss at the fundamental frequency. With more than 30 dB of power gain, the PA maintains to be unconditionally stable. The K-factor plot is illustrated in **Figure 14**. From DC to 5 GHz, K-Factor is more than 1. The power gain profile across output power is shown in **Figure 15**, which measures up to a maximum output power of 30.5 dBm, or 1.1 W. The 1 dB compression point of the PA is observed to be 29.5 dBm.

**Figure 16:**
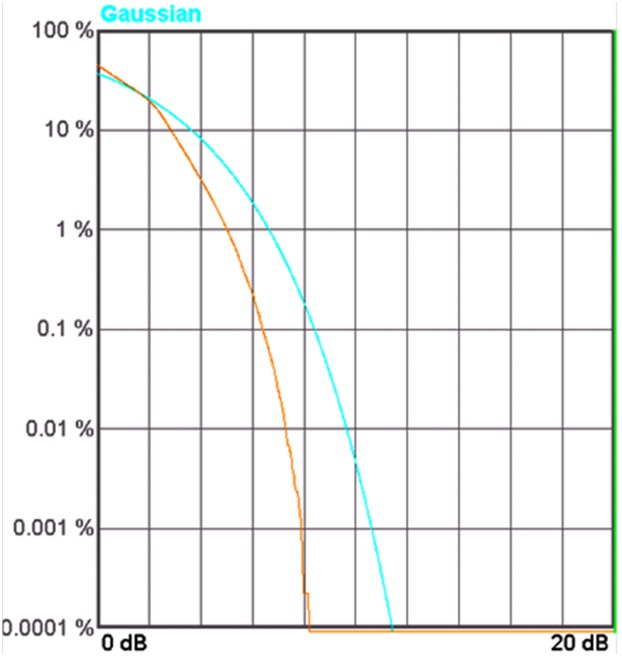
CCDF curve of the 20-FDMA multicarrier modulation scheme. At 0.001% the PAPR is 7.88 dB.

**Figure 17:**
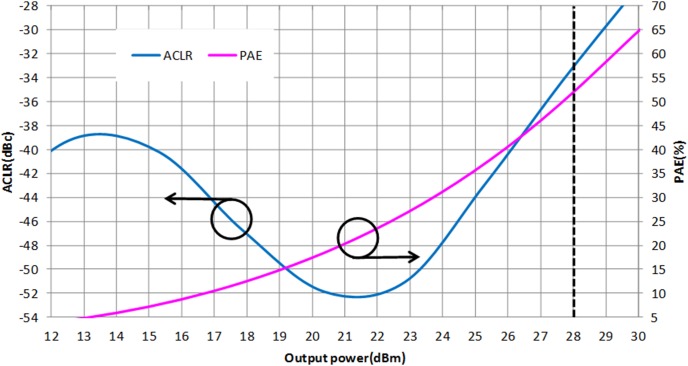
ACLR and PAE performances of the linearized PA at 1.95

**Figure 18:**
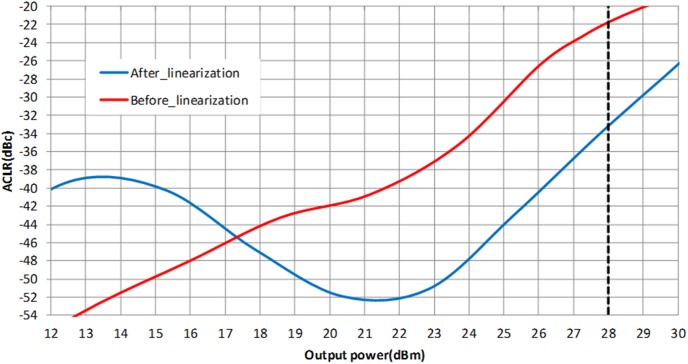
ACLR plot before and after linearization.

**Figure 19:**
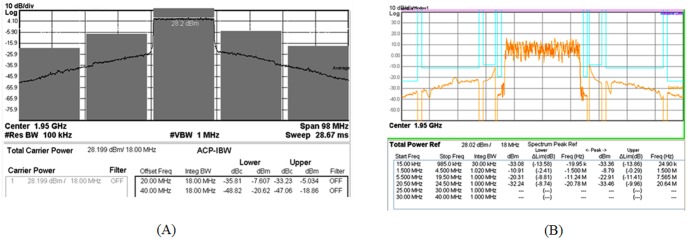
ACLR and spectral emission spectrum. (A) ACLR at output power of 28 dBm. (B) Measured spectrum, which is within the spectral mask at 28 dBm.

**Figure 20:**
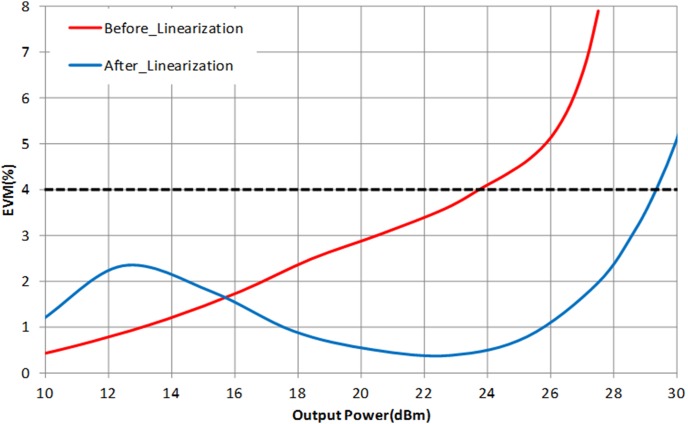
EVM plot of the linearized PA. The input signal is LTE 20

**Figure 21:**
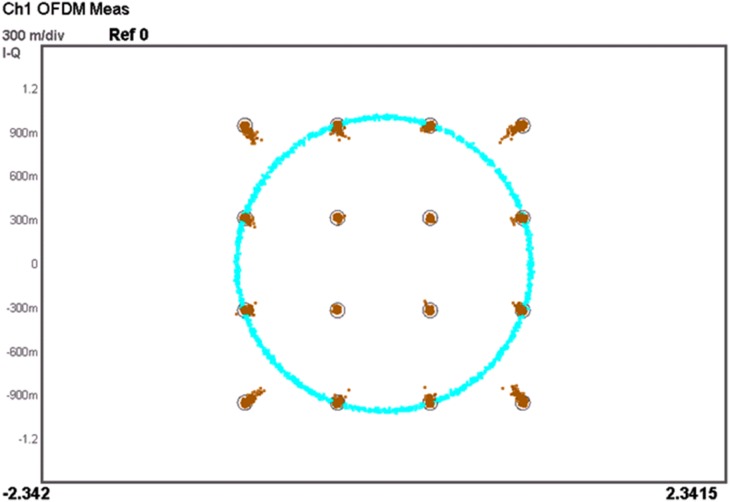
Constellation diagram at 28_out_.

For LTE operation, the designed PA is tested with a 16-QAM modulation signal which has 20 MHz channel bandwidth. The PAPR of the signal is 7.88 dB (at 0.001%), as illustrated in **Figure 16**. The resulting ACLR and PAE plot is shown in **Figure 17**. With a supply voltage of 3.3 V, the PA is capable to deliver PAE of 52.3% at output power of 28 dBm, with a corresponding ACLR reading out to −33.3 dBc. The specification for ACLR is −30 dBc, as per stated in the 3GPP specifications (3GPP TS 36.101), release 10.5 (2012). An extra margin of 3.3 dB in ACLR ensures PA works with comfortable headroom once integrated in the LTE transmitter system. **Figure 18** illustrates the ACLR of the PA before and after linearization. The IMD3 cancellation through the proposed predistorter circuit contributes to 11.3 dB improvement in ACLR at output power of 28 dBm. The higher ACLR at lower output power can be treated as a mild side effect due to injection of a distorted RF input signal to the input of the PA. Nevertheless, the ACLR at this power level is still below the specification. In **Figure 19**, the ACLR spectrum at the maximum linear output power of 28 dBm is illustrated. The measurement result shows that the linearized PA meets the ACLR specifications of −30 dBc and within the regulated spectral mask. **Figure 20** depicts the EVM plot. At the maximum linear output power of 28 dBm, the EVM achieved to be 2.36%, which is lower than the regulated 4% in specification. In relation to **Figure 20**, the plot of **Figure 21** gives the constellation point at 28 dBm of output power confirming the EVM performance of 2.36%.

**Table 1** tabulates the proposed PA’s measured performance summary. **Table 2** tabulates the performance comparison of the proposed PA with other reported works in this exact niche of application.

## Conclusion

A novel topology of wideband, high efficiency LTE power amplifier is presented in this work. The integrated analog pre-distorter (APD) provides a significant improvement in the ACLR and EVM performance at low backed-off output power. The integration has improved the efficiency of the PA at linear operating region as well. The proposed APD circuit does not jeopardize the input return loss, gain and stability of the PA, which are critical in constructing a reliable transmitter. At output power of 28 dBm, the PA delivers 52.3% PAE, confirming the ACLR and EVM specifications for wideband channel bandwidth of 20 MHz. Favorably, the size of the chip scales down to be less than 1 mm × 1 mm, which reduces the design cost. The results highlight the potential application of the proposed PA in a handset transmitter system, where it is capable to deliver higher linear output power at lower supply voltage as compared to the published works in table 2, thus prolonging the battery life.
